# Novel Low-Power Construction of Chaotic S-Box in Multilayer Perceptron

**DOI:** 10.3390/e24111552

**Published:** 2022-10-28

**Authors:** Runtao Ren, Jinqi Su, Ban Yang, Raymond Y. K. Lau, Qilei Liu

**Affiliations:** 1 School of Modern Post, Xi’an University of Posts and Telecommunications, Xi’an 710061, China; 2 School of Management and Economics, Xi’an University of Posts and Telecommunications, Xi’an 710061, China; 3 Department of Information Systems, City University of Hong Kong, Kowloon Tong, Hong Kong, China; 4 School of Cyberspace Security, Xi’an University of Posts and Telecommunications, Xi’an 710121, China

**Keywords:** S-box, multilayer perceptron, information theory, cyber security

## Abstract

Multilayer perceptron is composed of massive distributed neural processors interconnected. The nonlinear dynamic components in these processors expand the input data into a linear combination of synapses. However, the nonlinear mapping ability of original multilayer perceptron is limited when processing high complexity information. The introduction of more powerful nonlinear components (e.g., S-box) to multilayer perceptron can not only reinforce its information processing ability, but also enhance the overall security. Therefore, we combine the methods of cryptography and information theory to design a low-power chaotic S-box (LPC S-box) with entropy coding in the hidden layer to make the multilayer perceptron process information more efficiently and safely. In the performance test, our S-box architecture has good properties, which can effectively resist main known attacks (e.g., Berlekamp Massey-attack and Ronjom–Helleseth attack). This interdisciplinary work can attract more attention from academia and industry to the security of multilayer perceptron.

## 1. Introduction

**Figure 1:**
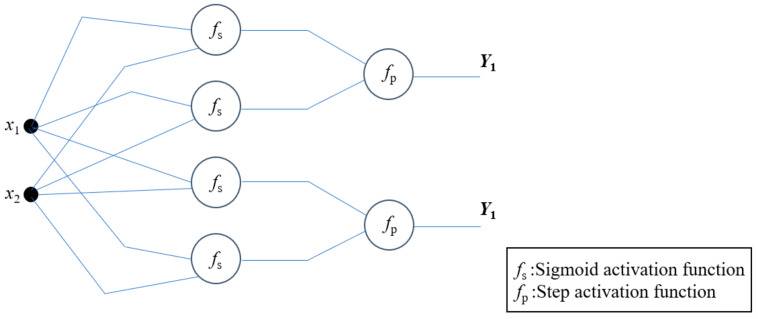
Structure of the LPC S-box in multilayer perceptron.

Multilayer perceptron (MLP) is a multilayer feedforward network model with one-way propagation [1,2,3]. Because of its high nonlinear mapping ability, MLP is one of the most basic network models in neural network research. From the perspective of information processing, MLP is an abstract simulation of biological neural networks to establish a simple biological neuron model. Therefore, the basic structure of MLP is based on the logic of biological neuron model. The most typical MLP includes three layers: input layer, hidden layer, and output layer (as shown in Figure 1). In the hidden layer, each node is equivalent to a perceptron, and each node represents a specific output function, which is called activation function [4]. The connection between each two nodes represents a weighted value for the signal passing through the connection, which is called the weight. The output of MLP will be different due to the difference of weight value and excitation function, and its powerful fitting ability can be used to solve more complex problems. MLP itself is usually the approximation of some algorithm or function in nature, and it may also be the expression of a logical strategy. With the gradual deepening of the research on MLP, it has great research potential in both theoretical research and application. At present, MLP has been applied in many commercial and industrial fields and has brought varying degrees of productivity improvement (e.g., pattern recognition, function approximation, and optimal prediction) [5,6,7].

The interior of multilayer perceptron is a highly nonlinear information processing system composed of multilayer single perceptron interconnection [8]. When neurons are in two different states of activation or inhibition, it can be called a nonlinear relationship. The network composed of neurons with a threshold has better performance, which can improve fault tolerance and storage capacity. Each neuron of the multilayer perceptron receives the input of a large number of other neurons, and generates the output through the parallel network, affecting other neurons. This mutual restriction and interaction between the networks realizes the nonlinear mapping from the input state to the output state space [9,10]. However, the overall performance of multilayer perceptron is not the superposition of the performance of local neurons, and its nonlinear mapping ability is limited. Therefore, when processing information with higher complexity, we can introduce stronger nonlinear components (e.g., S-box) to make up for this defect. As a nonlinear component of multilayer perceptron, S-box is also an important part of symmetric cipher (e.g., block cipher) [11,12,13].

The higher dimension multilayer perceptron has very complex nonlinear dynamic behaviors, which contain various functions (e.g., activation function and step function). As Piotr et al. remarked [14], securely enhance multilayer perceptron must satisfy certain conditions, the higher the dimension of S-box, the more statistical analysis it can be applied to multilayer perceptron, which is complex for algorithm designers and malicious password analysts. Yet, the carefully designed S-box is just a strong nonlinear Boolean (vector) map with avalanche characteristics. This means, in fact, that the S-box can be considered as a black box that transforms any input vector into a balanced vector in a non-predictable way (i.e., nonlinear). Figure 1 shows the architecture of different component combinations to achieve balance.

At present the methods of S-boxes applied on multilayer perception for improving security complexity are based on two parallel methodologies [15,16]. The first one mainly uses mathematical theories and statistical investigations, while the other is additionally supported by the practitioner’s experience. To second a link between the both methodologies we propose design a structure of S-box based on multilayer perceptron to process the data from the overall point of view, this method can enhance its information processing ability and improve the overall security.

Accordingly, for the complex nonlinear architecture of multilayer perceptron, if the computational cost of data itself (e.g., compressed data) is reduced, its nonlinear mapping ability can also be improved. Modern lossless data compression program is realized through the combination of general compression technology and entropy coding. The role of lossless data compression algorithm is to increase the randomness of data (i.e., increase entropy). On the other hand, the entropy coding method compresses an original data unit into the minimum code as the compressed data. Although each of these technologies can achieve the compression effect, the combination will produce a better compression ratio. Then, they maximize the entropy of the compressed data (such as arithmetic coding and Hoffman coding). Therefore, in the hidden layer, compression technology and entropy coding can be combined to increase the throughput of the multilayer perceptron, reduce redundant information, and reduce computational overhead.

Based on the above factors, we combine the methods of cryptography and information theory to introduce S-box into multilayer perceptron with Levenshtein entropy coding-based in the hidden layer to make the multilayer perceptron process information more efficiently and safely.

In previous studies, there is a scheme to apply S-box to multilayer perceptron. Arrañaga et al. proposed a scheme that S-box can apply it to multilayer perceptron [17]. But it does not test the cryptographic characteristics of the implemented S-box, nor is it applied to the replacement of image processing.

Zhu et al. [18] proposed an improved chaotic map for image encryption system. The framework preliminarily analyzes the conventional technologies of one-dimensional chaotic system, replacement box production structure and image encryption algorithm. The double chaotic S-box algorithm includes forward backward confusion diffusion operation to enhance the performance of image encryption system. The shortcomings of the proposed model do not discuss the side channel attack in the encryption framework, nor is it applied to multilayer perceptron.

A.S. et al. [19] involved an S-box with hybrid prediction and adaptive chaos, and calculated and analyzed various performance parameters. However, it is based on embedded system rather than multilayer perceptron, which makes image processing less obvious.

Yang et al. [20] designed a new chaotic S-box diffusion method based on 2d-mccm, which improved the security and efficiency, and proposed a new image encryption algorithm, but it was not constructed based on multilayer perceptron.

Zhang et al. [21] introduced the learning algorithm based on multilayer perceptron and S-box to improve the security integrity of the system. The proposal uses the new way of approach to decompose the huge input into two equal parts and each part is trained by two perceptrons combined with a special 172P perceptron. It also convenient to quickly train the weight-threshold values of the Boolean function in the network through DNA-like learning algorithm.

Kotlarz and Kotulski [22] discussed the use of S-boxes to implement cryptographic schemes in multilayer perceptron. To realize the elementary permutation, 2-bit and 3-bit block of bits were transformed into a block of bits as a combination of small blocks (i.e., multilayer perceptron). The permutation of 16-bit blocks is realized in this model. The advantage of this method is that it makes use of the fragmentary training sets for each block, at the server side, and the complete training set for the whole multilayer perception in order to realize the cryptographic algorithm. This fragmentary training system gives some security for the algorithm update process provided the internal structure of the complete topology remains secret.

For the above research, their research scheme not only failed to study the S-box through Boolean function, resulting in the inability to analyze the cryptographic properties, but also failed to combine the S-box and multilayer perceptron into image processing.

**Figure 2:**
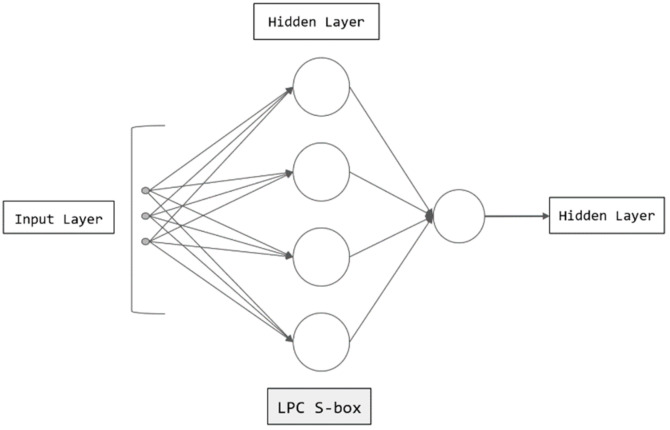
Structure of the LPC S-box in multilayer perceptron.

For the above research, their research scheme not only failed to study the S-box through Boolean function, resulting in the inability to analyze the cryptographic properties, but also failed to combine the S-box and multilayer perceptron into image processing. We give a low-power chaotic S-box based on multilayer perceptron to optimize, and apply it to image processing to improve its information processing ability in the multilayer perceptron. The application of LPC S-box in multilayer perceptron is shown in Figure 2. The contributions of this article are as follows:

(1) We combine the methods of cryptography and information theory to design a low-power chaotic S-box, and carry out noise reduction and entropy coding compression in the hidden layer, so that the multilayer perceptron can process images more efficiently and safely.
(2) Our S-box has the function of replacement, which aims to confuse the binary string after encoding and compression, prevent the computer from being invaded illegally and maliciously decode the image, so as to encode the image that is difficult to be processed by the computer and has low processing efficiency efficiently and safely.
(3) We selected a group of S-boxes with good cryptographic performance for performance testing. The results show that our scheme can effectively resist algebraic attacks, DPA attacks, etc., which can not only make up for the limited nonlinear mapping ability of multilayer perceptron, but also improve the security of multilayer perceptron model.

## 2. Preliminaries

### 2.1. Boolean Function

Let *n* and *m* be two positive integers, and the vector space $F_{2}^{n}\to F_{2}^{m}$ mapping is called (*n*, *m*) function (i.e., multi output Boolean function or vector Boolean function), where *n* is the multi-input and *m* is the multi-output. When *m* = 1, it can be called a single output Boolean function. When *m* = *n*, we call this multi output Boolean function S-box.

### 2.2. Algebraic Degree

Let *F*(*x*) be a (*n*, *m*) function, then the algebraic degree of *F*(*x*) is defined as:

$$deg\;F=\underset{\;}{\min}\{deg\left(v\cdot F\right)|0\neq v\in F_{2}^{m}\}$$

where v·F is called the component function, and the algebraic degree of the multi output Boolean function is the minimum value of the non-zero linear combination of all its component functions.

### 2.3. Algebraic Immunity

The algebraic immunity of n-ary Boolean function *F* is expressed by *AI* (*F*) and is defined as:

$$AI\left(F\right)=min\{deg\;g|0\neq Ann\left(F\right)\mathrm{or}\;Ann\left(1+F\right)\}$$

where $Ann\left(F\right)=\{g\;|g\epsilon B_{n},Fg=0\}$, *G* is called the annihilator of Boolean function *F.*

### 2.4. Differential Uniformity

Let *F*(*x*) be a (*n*, *m*) function, then the differential uniformity of *F*(*x*) is:

$$\delta_{F}=\underset{0\neq \alpha \epsilon F_{2}^{n}}{\max}\underset{\beta \epsilon F_{2}^{m}}{\max}\left|\{x\epsilon F_{2}^{n}\right|F\left(x+\alpha \right)-F\left(x\right)=\beta \}|$$

The difference uniformity of *F*(*x*) satisfies $2^{n-m}\leq \delta_{F}\leq 2^{n}$, if it $\delta_{F}$ is the maximum value $2^{n}$, *F*(*x*) is an affine function, α and β $\in F_{2}^{n}$, *n* is the multi-input, *m* is the multi-output. If $\delta_{F}$ is the minimum value $2^{n-m}$, it is called a fully nonlinear function.

## 3. Methodology

**Figure 3:**
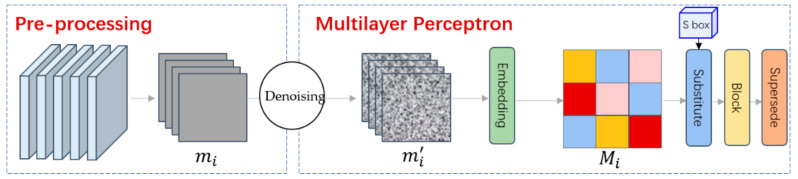
Interaction of our scheme.

In this paper, a lightweight chaotic S-box is constructed based on entropy coded images and embedded in multiple hidden layers of multilayer perceptron. The application of chaotic S-box in multilayer perceptron is shown in the figure. The chaotic S-box based on entropy coded images is an efficient and safe construction scheme. Figure 3 shows the interaction of our scheme.

Our scheme includes the following algorithms: pre-processing, denoising, embedding, substitute, block, and supersede. The specific definition of each algorithm is as follows:

(1) Pre-processing: first, the original image is processed at the input layer of the multilayer perceptron, and a series of binary sequences are obtained through specific algorithms.
(2) Denoising: in the first hidden layer, the pixels of the input image are filtered to obtain a noise reduction image with redundant information removed.
(3) Embedding: the algorithm performs Levenshtein entropy coding based on each image and completes weighting to obtain a compressed image in the second hidden layer.
(4) Substitute: this algorithm converts the image into a one-dimensional sequence by generating the initial parameters of chaos. The blurred image can be obtained by shifting the image according to the sequence.
(5) Block: grouping the long sequence after replacement.
(6) Supersede: the operation is to input the one-dimensional sequence into the S-box sequentially to obtain a new sequence.

## 4. Syntax

### 4.1. Pre-Processing

This algorithm runs by the input layer of deep feed-forward artificial neural network. The deep structure comprises many layers of non-linearly activating nodes. Each neuron-like node is connected from one layer to another. The input of one layer is connected to another layer with different adjustable weights to form a complete neural network. When the original image is input to the deep feed-forward artificial neural network, it will be extracted by equation $W\left(t\right)=\sum_{i=1}^{n}m_{i}\gamma_{1}+d$, where $W\left(t\right)$ is the activity of the neurons in the input layer at a time *t*, $m_{i}$ denotes the input *M* × *N* size image with adjustable weight, $\gamma_{1}$ is the weights among the input and hidden layer, and d represents the bias. The following Algorithm 1 describes the implementation process of Pre-Processing.
**Algorithm 1** Pre-processing.1:**Input**: $m_{i}$2:**Output**: $W\left(t\right)$3:extract features $W\left(t\right)=\sum_{i=1}^{n}m_{i}\gamma_{1}+d$4:**Return** the activity of the neurons $W\left(t\right)$

### 4.2. Denoising

This algorithm is executed by the first hidden layer. Its purpose is to improve the image contrast and eliminate the unwanted pixels in an image. In this algorithm, the adaptive sigma filter (smoothing filter) places the pixels of the input image in the adaptive kernel (i.e., window) with different sizes. Then, it organizes the neighboring pixels $V_{n}$ (i.e., $V_{i,j-1}$, $V_{i-1,j}$, $V_{i,j+1}$, $V_{i+1,j}$) in the form of a matrix with ‘*i*’ row and ‘*j*’ column. In the kernel area, the intensity values of pixels are sorted in ascending order. Finally, the noise pixels are removed from the filter window by the central pixel $V_{i,j}$ to obtain the denoised image $m_{i}^{\prime }$. The following Algorithm 2 describes the implementation process of Denoising.
**Algorithm 2** Denoising.1:**Input**: $m_{i}$2:**Output**: $m_{i}^{\prime }$3:divides the original picture $m_{i}$ into segments;4://segments are horizontal and vertical;5:arranges the pixel in a kernel size R*R; 6:picks the central pixels $V_{i,j}$;7:picks the neighboring pixels $V_{n}$;8:measures the deviation $\tau_{ij}=\sum \left|V_{i,j}-V_{n}\right|$;9:removes the noisy pixels from the filter window;10:**Return** the denoised image $m_{i}^{\prime }$


### 4.3. Embedding

This algorithm (i.e., Levenshtein entropy encoding-based compression) is run by the second hidden layer. Embedding can make pictures lossless compression, reduce storage costs, and improve transmission efficiency. For this algorithm, each image is weighted by $\omega =\rho (m_{i}^{\prime })$, where ρ indicates a weight assigned to $m_{i}^{\prime }$, the sum of weight value is equal to one (ρ = 1), and the resultant code is termed a complete code. The length of codeword and the weighted path length are determined. Then, the probability and entropy are calculated. Finally, the compressed images are obtained at the second hidden layer by the output of previous layer $B\left(t\right)$ and the output of deep learning $O\left(t\right)$. The following Algorithm 3 describes the implementation process of Embedding.
**Algorithm 3** Embedding.1:**Input**: $m_{i}^{\prime }$2:**Output**: $M_{i}$3:assigns the weight $\omega =\rho (m_{i}^{\prime })$; 4:sets the length of codeword $C\left(w\right)=\left(\psi_{1},\ldots ,\psi_{n}\right)$;5:sets the weighted path length $\omega_{n}=\rho l_{c}$;6://$l_{c}$ is the lengths of the code words7:calculates the probability of pixels $P=2^{-l_{c}}$;8:calculates the entropy $H\left(E\right)=-\rho {log}_{2}\rho$;9:calculates $B\left(t\right)=\sum_{i=1}^{n}m_{i}^{\prime }a_{1}+a_{2}b\left(t-1\right)$;10:calculates $O\left(t\right)=B\left(t\right)a_{3}$;11://$a_{1}$ is the weight of hidden layers;12://$a_{2}$ is a weight between input and hidden layers;13://$a_{3}$ is an adjustable weight of two layers;14://$b\left(t-1\right)$ is the output from first hidden layer;15:**Return** the compressed image $M_{i}$

### 4.4. Substitute

The algorithm first generates the secret key of 256 bit binary number, and then the secret key will be divided into 32 8-bit binary numbers $k_{i}\left(i=1,2\cdots ,32\right)$. Next, the system generates the initial parameters (*w_0_*, *e*, *q*, *x_0_*, *v_1_*, *v_2_*) of chaos, converts the picture into a one-dimensional sequence *D* with the size of *M* × *N*, and then substitutes the chaotic parameters into the formula: $w_{n+1}=1-q\left|ew_{n}\left(1-w_{n}\right)-\left(1/q\right)\right|$, where $w_{n}\in \left[0,1\right]$, $e\in \left[0,2\right]$, $q\in \left[0,4\right]$, *e* and *q* are system parameters. The sequence *T* with the length of *M* × *N* can be obtained by the above formula for eliminating the transient benefits. Then, the substitution operation is carried out. Sequence *T′* is obtained by arranging the values in sequence *T* from small to large. Displacement sequence *T″* can be obtained according to the position information of the elements of sequence *T′* in sequence *T*. Finally, image ${M_{i}}^{\prime }$ can be obtained by displacement of image $M_{i}$ according to the sequence. The following Algorithm 4 describes the implementation process of Substitute.
**Algorithm 4** Substitute.1:**Input**: $M_{i}$2:**Output**: ${M_{i}}^{\prime }$3:generates the $w_{0}=\frac{\left(k_{1}⊕k_{2}⊕k_{3}⊕k_{4}⊕k_{5}⊕k_{6}\right)+\sum_{i=1}^{32}k_{i}}{2^{8}}mod\left(1\right)$;4:generates the $q=\frac{\left(k_{6}⊕k_{7}⊕k_{8}⊕k_{9}⊕k_{10}⊕k_{11}\right)+\sum_{i=1}^{32}k_{i}}{2^{8}}mod\left(1\right)+1$;5:generates the $e=\frac{\left(k_{11}⊕k_{12}⊕k_{13}⊕k_{14}⊕k_{15}⊕k_{16}\right)+\sum_{i=1}^{32}k_{i}}{2^{8}}mod\left(1\right)+2$;6:generates the $x_{0}=\frac{\left(k_{17}⊕k_{18}⊕k_{19}⊕k_{20}⊕k_{21}⊕k_{22}\right)+\sum_{i=1}^{32}k_{i}}{2^{8}}mod\left(1\right)$;7:generates the $v_{1}=\frac{\left(k_{22}⊕k_{23}⊕k_{24}⊕k_{25}⊕k_{26}⊕k_{27}\right)+\sum_{i=1}^{32}k_{i}}{2^{8}}mod\left(1\right)+3$;8:generates the $v_{2}=\frac{\left(k_{27}⊕k_{28}⊕k_{29}⊕k_{30}⊕k_{31}⊕k_{32}\right)+\sum_{i=1}^{32}k_{i}}{2^{8}}mod\left(1\right)+3$;9://$l_{c}$ is the lengths of the code words10:displaces the $M_{i}$ to one-dimensional sequence D;11:eliminates transient effects to get sequence T;12:executes substitution operation;13:**Return** the image ${M_{i}}^{\prime }$

### 4.5. Block

The block algorithm is to segment the image to facilitate the next supersede algorithm. The following Algorithm 5 describes the implementation process of Block.
**Algorithm 5** Block.1:**Input**: ${M_{i}}^{\prime }$2:**Output**: ${A_{i}}^{\prime }$3:divides the ${M_{i}}^{\prime }$ to *m* × *n* numbers block with *M*/*m* × *N*/*n* size;4:converts pixels of each block into one-dimensional sequence $A_{i}$;5:**Return** the sequence $A_{i}$


### 4.6. Supersede

The specific process of this S-box operation is to input the one-dimensional sequence $A_{i}$ into the S-box in turn to obtain the sequence $B_{i}$. The following Algorithm 6 describes the implementation process of Supersede.
**Algorithm 6** Supersede.1:**Input**: $A_{i}$2:**Output**: $B_{i}$3:run equation $S\left(B_{i}\right)=A_{i},\left(i=1,2,\cdots ,m\times n\right)$;4:**Return** the sequence $B_{i}$


## 5. Benchmark Test

In this section, we estimate the index performance of our S-box and other schemes. Experimental environment for performance analysis is as follows: the processor is Intel^®^ Core^TM^ i5-8300H CPU @2.30 GHz; the system type is a 64-bit operating system. Based on this system, this paper uses C programming language to calculate nonlinearity, differential uniformity, and transparency order operations.

In the multilayer perceptron, a series of operations of S-box can be used in the hidden layer. Because the S-box has a high degree of nonlinearity, it is related to the nonlinear mapping of the hidden layer. The nonlinearity, difference uniformity, and transparency order involved in this paper can be applied to the adaptive function of multilayer perceptron, which plays an important role in the optimization of S-box. The relation of attributes of S-box based on multilayer perceptron is shown in Table 1 below.

We classify all the optimal 4-bit S-boxes and generate 16 optimal S-boxes under different nonlinearity and uniformity conditions according to the affine equivalence principle (i.e., optimum in differential, linear, and algebraic attacks). In this paper, a new scheme of S-box with multilayer perceptron based on Levenshtein entropy coding algorithm is proposed, and the performance of various scheme 4-bit S-box is tested.

Ta Thi Kim Hue et al. [23] defined the 4-bit optimal S-box for the first time, that is, the bijective S-box whose nonlinearity and differential uniformity reach the critical value 4 at the same time. We have found the representative elements of eight types of optimal 4-bit S-boxes and used intuitive symbols $G_{i}$ to represent different schemes. The optimal 4-bit S-box of our S-box is named $G_{0,1,2,3,4,5,6,7}$. The 4-bit S-box proposed by Canteaut et al. [24] named $G_{8,9}$ The data of ours 8 optimal 4-bit S-box and Canteaut et al. S-boxes [24] are shown in Table 2.

### 5.1. Nonlinearity

**Figure 4:**
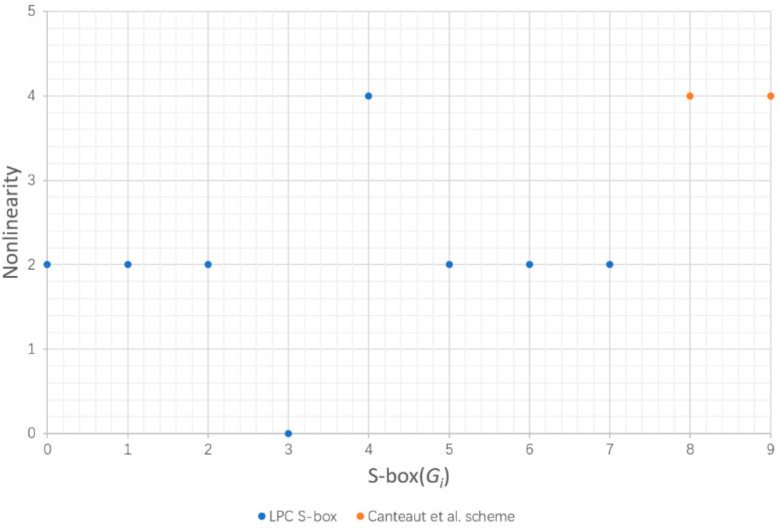
Nonlinearity of each scheme [24].

In order to resist linear cryptographic attacks, Boolean functions used in cryptosystems should be as far away from the Hamming distance of all affine functions as possible [25,26]. The nonlinearity *NL*(*F*) of Boolean function *F* is defined as the minimum Hamming distance between *F* and all affine functions. The nonlinearity of each scheme is obtained from the data input into the S-box, as in Figure 4.

The upper bound of the nonlinearity of a n × n S-box is $2^{n-1}-2^{\frac{n}{2}-1}$, and the upper bound of the nonlinearity of a 4-bit S-box is 6. It can be seen from Figure 4 that the nonlinearity of LPC S-box is up to 4, which is high for 4-bit, because it is difficult to construct an S-box close to the upper bound. The higher the nonlinearity, the closer the nonlinear ability between input and output is to the upper bound, the stronger the corresponding anti-linear attack ability. Hence, LPC S-box has excellent ability to resist linear cryptographic attacks.

### 5.2. Differential Uniformity

**Figure 5:**
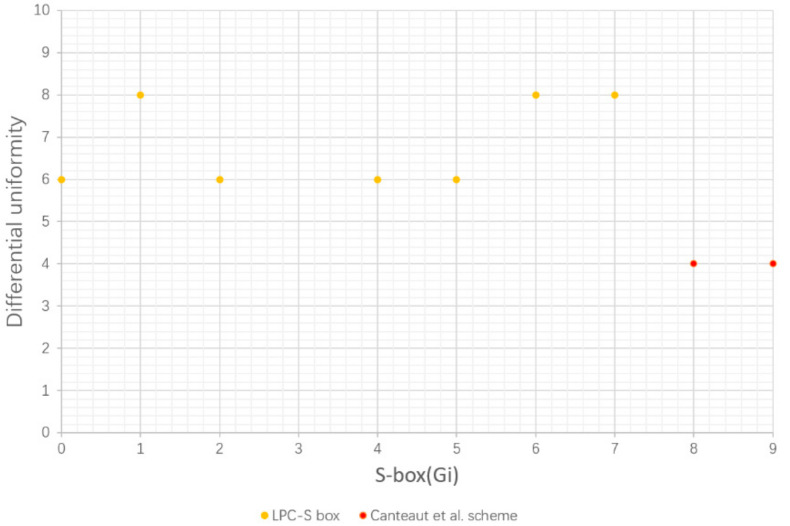
Differential uniformity [24].

The value of differential evenness of Boolean function is inversely related to the ability to resist differential cryptographic attacks. The differential uniformity of each scheme is obtained from the data input into the S-box, as in Figure 5.

The range of differential uniformity for n × n S-boxes is $0\leq \delta_{F}\leq 2^{n}$, and the range of differential uniformity for 4-bit S-boxes is $0\leq \delta_{F}\leq 16$. By comparison, the difference uniformity of the LPC S-box is up to 8, and the difference uniformity of the Canteaut et al. [24] scheme is all 4. In terms of security performance, the smaller the maximum value of the differential propagation probability, the stronger the S-box resists differential attacks.

Hence, LPC S-box has excellent ability to resist differential attacks.

### 5.3. Transparency Order

**Figure 6:**
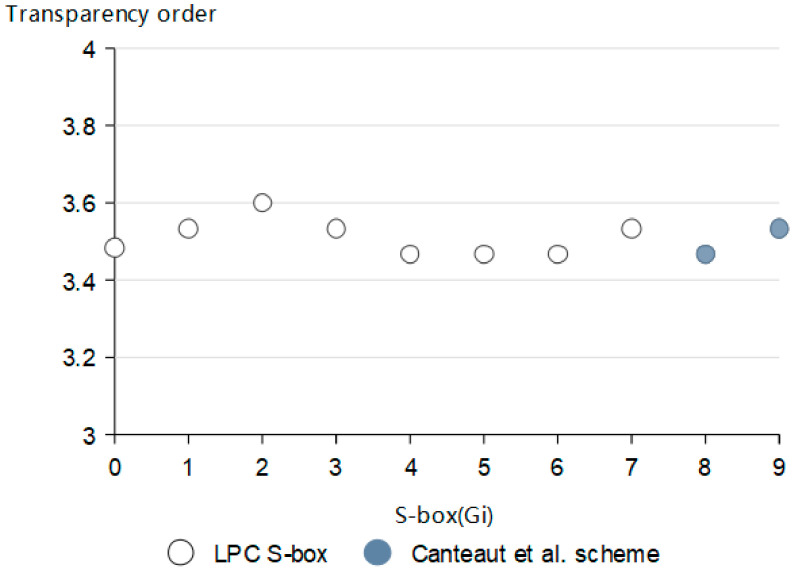
Transparency order of each scheme [24].

Transparency order is an indicator to measure the ability of Boolean functions to resist differential power analysis (DPA) attacks. The lower the transparency order of Boolean function, the stronger the ability to resist DPA attack. The DPA attack has nothing to do with the security of the algorithm, which is an attack based on the characteristics of the algorithm on the device [27]. The transparency order of each scheme is obtained from the data input into the S-box, as in Figure 6.

According to the above definition analysis, the transparency order, and nonlinearity of the 4-bit S-box are negatively correlated. When the S-box has high nonlinearity, its transparency order is low. It can be seen from Figure 6 that the average level of transparency order of LPC S-box is slightly smaller than that of the Canteaut et al. [24] scheme. Correspondingly, LPC S-box has excellent ability to resist DPA attacks.

### 5.4. Algebraic Degree

**Figure 7:**
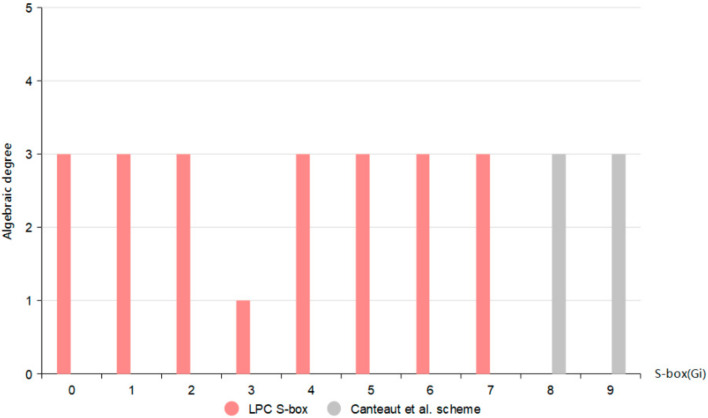
Algebraic degree of each scheme [24].

For resisting the Berlekamp Massey-attack and the Ronjom-Helleseth attack, the Boolean function in the S-box must have a high algebraic degree. The algebraic degree of function *F*, expressed by deg(F), is the number of variables contained in the highest order term in its algebraic normal form. We obtain their algebraic degree by inputting the data of S-box, and obtain the data shown in Figure 7 according to the simulation calculation.

The upper bound for the number of algebras of the 4-bit S-box is 4, and it can be seen from the figure that the number of algebras of the LPC S-box is up to 3. This has a higher algebraic degree for a 4-bit S-box, because it is difficult to construct an S-box close to the upper bound, and the closer the algebraic number is to the upper bound, the stronger the anti-algebraic attack capability of the corresponding S-box. Therefore, both the LPC S-box and the Canteaut et al. [24] scheme have outstanding resistance to Berlekamp Massey-attack and the Ronjom-Helleseth attack.

### 5.5. Algebraic Immunity

**Figure 8:**
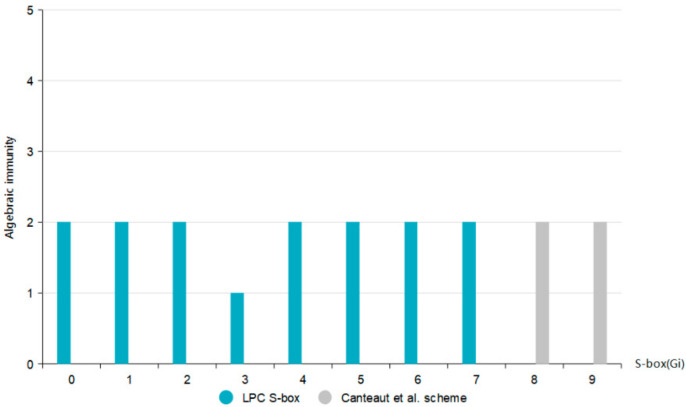
Algebraic immunity of each scheme [24].

Algebraic attacks are often used in stream cipher systems, threatening the security of the entire cryptosystem. In order to resist algebraic attacks, people have proposed a new security index of Boolean functions (e.g., algebraic immunity). The algebraic immunity of n-ary Boolean function *F* is expressed by AI(F). We obtain their algebraic immunity by inputting the data of S-box, and obtain the data shown in Figure 8 according to the simulation calculation.

The upper bound of the algebraic immunity of an n × n S-box is $AI\left(F\right)\leq [\frac{n}{2}]$, and the upper bound of the algebraic immunity of a 4-bit S-box is 2. As can be seen from Figure 7, the algebraic immunity of LPC S-boxes all reach the upper bound of 2, which has the highest algebraic immunity for 4-bit S-boxes, so the constructed S-boxes are optimal. Hence, LPC S-box has excellent ability to resist algebraic attacks.

### 5.6. Overall Performance Analysis

In the benchmark test, the cryptographic performance index of the S-box of the existing scheme is compared, which is based on the verification of the security of the S-box in the multilayer perceptron. The nonlinearity, transparency order, algebraic degree, and algebraic immunity selected in the experiment are supplementary explanations for the security of the S-box. The data itself reflects the security of the 4-bit S-box. The calculation results of the transparency order verify that the correlation between the original data and the output data is reduced through the S-box of the hidden layer, and a better anti-DPA performance is obtained. Related research has proved that power consumption is the main function of Hamming weight of data privacy in displacement operation [28]. If Hamming weight of data privacy is disclosed, attackers can determine the number of bits of data privacy by solving a series of linear equations. In DPA, the attacker associates the power consumption with the data value manipulated in the replacement process, and uses the corresponding function solution to obtain data privacy information. Therefore, a high-performance transparency order will reduce power consumption. High nonlinearity is also difficult for attackers to solve linear equations [29], so the calculation of nonlinearity can also show the correlation between S-box and low power consumption. Therefore, we tested based on the cryptographic performance indicators of the S-box in the multilayer perceptron, and proved that LPC S-box reduced the overhead for the overall system operation in terms of computing, thus achieving the goal of low power consumption.

## 6. Summary

In this paper, a low-power chaotic S-box is designed based on the multilayer perceptron, and the existing image is preprocessed, denoised, entropy coded compression, S-box replacement, and other operations through the algorithm to make the image processing more efficient and safe. By comparing the performance of eight types of low-power 4-bit S-boxes with that of some commonly used lightweight 4-bit S-boxes, we know that the LPC S-box has excellent performance. In the performance test of Boolean functions in low-power S-boxes, LPC S-box has good nonlinearity, differential uniformity, and transparency, and can effectively resist linear attacks, differential attacks, and DPA attacks.

## Figures and Tables

**Table 1:** Relation.

Multilayer perceptron	S-box
Adaptability	Reduce manual intervention
Fault tolerance	Nonlinearity
Weight coefficient	Differential uniformity
Threshold coefficient	Transparency order
Iterative training	Multi turn transformation
Ergodicity	Confusion principle
Stability	Reliability
Low-power	Differential power analysis

**Table 2:** The 4-bit S-box representation.

Scheme	S-Box	Representative Element
LPC S-box	$S_{0}$	0, 2, 10, 3, 4, 6, 9, 14, 11, 7, 5, 1, 12, 8, 13, 15
	$S_{1}$	6, 10, 14, 2, 15, 8, 13, 1, 12, 9, 7, 4, 5, 0, 3, 11
	$S_{2}$	2, 1, 6, 12, 4, 10, 15, 7, 3, 5, 13, 11, 9, 8, 14, 0
	$S_{3}$	4, 8, 3, 15, 11, 7, 12, 0, 9, 5, 14, 2, 6, 10, 1, 13
	$S_{4}$	10, 13, 4, 5, 7, 3, 9, 12, 14, 6, 0, 15, 8, 1, 2, 11
	$S_{5}$	3, 5, 8, 2, 13, 4, 12, 6, 7, 0, 9, 10, 15, 11, 1, 14
	$S_{6}$	9, 13, 8, 11, 3, 15, 5, 0, 14, 7, 1, 4, 12, 10, 2, 6
	$S_{7}$	9, 12, 15, 0, 1, 8, 2, 11, 3, 14, 13, 4, 5, 10, 6, 7
Canteaut et al. [24]	$S_{8}$	0, 6, 14, 1, 15, 4, 7, 13, 9, 8, 12, 5, 2, 10, 3, 11
	$S_{9}$	0, 9, 13, 2, 15, 1, 11, 7, 6, 4, 5, 3, 8, 12, 10, 14

## Data Availability

The data used to support the findings of this study are included within the article.
